# Spotlight on borderline-IGHV mutational status in chronic lymphocytic leukemia

**DOI:** 10.3389/fonc.2024.1430225

**Published:** 2024-06-06

**Authors:** Souraya Rammal, Warde Semaan, Natalia Aprahamian, Romy Moussallem, Alain Chebly

**Affiliations:** ^1^ Faculty of Medicine, Saint Joseph University, Beirut, Lebanon; ^2^ Center Jacques Loiselet for Medical Genetics and Genomics (CGGM), Faculty of Medicine, Saint Joseph University, Beirut, Lebanon

**Keywords:** chronic lymphocytic leukemia, CLL, immunoglobulin heavy chain gene, IGHV, prognosis, biomarkers, Lebanon

## Abstract

Mutated or unmutated immunoglobulin heavy chain (*IGHV)* gene is an important prognostic factor in chronic lymphocytic leukemia (CLL). However, a small fraction of patients with CLL are classified as borderline (BL)-IGHV. Few data are available on this subgroup of CLL. In this paper, we retrospectively report and analyze data from 21 patients with BL-IGHV CLL, showing the heterogeneity of this subgroup of CLL and paving the way for more research focusing on this entity to optimize the management and treatment of patients with Borderline-IGHV CLL.

## Introduction

Chronic lymphocytic leukemia (CLL) is a mature B-cell neoplasm characterized by aberrant accumulation of monoclonal lymphocytes. CLL Patients can display different clinical outcomes (1). Today, prognostic stratification is based on the presence of cytogenetic alterations such as deletion(17p), del(11q), del(13q) and trisomy 12, in addition to molecular biomarkers, including the mutational status of the immunoglobulin heavy chain gene (*IGHV)* (2, 3). Indeed, the presence and load of somatic hypermutation within the rearranged *IGHV* gene of the B-cell receptor (BCR) classifies CLL into two main categories: Unmutated CLL (U-CLL) and mutated CLL (M-CLL). Classification into M-CLL or U-CLL is based on the established cutoff value (98%) for the identity to the germ line: U-CLL >98% and M-CLL <98% (4). However, for cases close to the cutoff, showing homology between 97 and 97.99%, results should be interpreted with caution due to the prognostic implications. This emerging “third group” is named borderline *IGHV* (BL-IGHV) and raised interpretation issues whether these patients should be classified as M-CLL cases or not (5). Recently, BL-IGHV cases are receiving more attention, as the clinical outcome of this group remains poorly defined.

Patients with BL-IGHV represent a small fraction of CLL patients, reported to be around 5% only, and there is a lack of data on this subgroup of CLL in the literature. Only few studies focusing on the outcome of this subgroup have been published, and yet they show discordant data and several discrepancies, especially when considering BL-IGHV as M-IGHV or U-IGHV (5–7). In a recent paper, Angotzi et al. reported data on 30 BL-CLL cases showing similarities and discrepancies with previously published studies, and yet they were unable to resolve the outcome of BL-IGHV CLL patients (8).

In a previous work, our team reported data on CLL genetic markers in a large series of Lebanese patients (312 patients), including the *IGHV* mutational status (9). The evaluation of the *IGHV* mutational status was performed in accordance with the European Research Initiative on CLL (ERIC) recommendations (4). Several conclusions were drawn about the similarities and differences compared to previously published data. However, the BL-IGHV entity was not evaluated solely in the previous study. Here, we retrospectively analyzed data from CLL patients with BL-IGHV referred to the Center Jacques Loiselet for Medical Genetics and Genomics (CGGM) at Saint Joseph University of Beirut, a tertiary referral center in Lebanon, between 2018 and 2024.

## BL-IGHV CLL cohort

Among a total of 424 CLL cases analyzed, only 21 BL-IGHV were detected (4.95%), which perfectly aligns with the 5% fraction defining the BL-IGHV group. The patients showed a homology to the germ line between 97.19% and 97.92%. Among these patients, 12 were males (57%) and 9 were females (43%) (**Table 1**), which is quite similar to what is published about CLL in Lebanon, mutated and unmutated together. The median age at the time of diagnosis was 63 years (mean=64), which is slightly lower than the median age of 67 years reported in all CLL groups in Lebanon (9).

**Table 1:** Demographic characteristics and genetic results of the 21 BL-IGHV CLL patients (n=21).

Patient	Age (Years)	Sex	IGHV family	Percentage of homology	#CLL subset*	FISH result
1	63	F	IGHV3–33	97.57%	Not Classified	del(13q) monoallelic
2	60	M	IGHV4–39	97.59%	Not Classified	N
3	55	F	IGHV3–30	97.92%	Not Classified	N
4	48	F	IGHV4–34	97.89%	Not Classified	N
5	66	M	IGHV4–34	97.89%	Not Classified	N
6	85	M	IGHV3–49	97.28%	Not Classified	del(13q) and del(11q)
7	58	F	IGHV1–2	97.92%	Not Classified	N
8	75	F	IGHV3–74	97.92%	Not Classified	del(13q) and del(17p)
9	69	M	IGHV4–61	97.25%	Not Classified	del(13q) biallelic
10	74	M	IGHV1–2	97.57%	Not Classified	del(13q) monoallelic
11	75	M	IGHV3–7	97.57%	Not Classified	N
12	75	M	IGHV4–39	97.94%	Not Classified	del(13q) and del(17p)
13	53	M	IGHV1–18	97.92%	Not Classified	N
14	52	F	IGHV5–51	97.22%	Not Classified	N
15	59	M	IGHV4–34	97.19%	Not Classified	del(13q) monoallelic
16	86	F	IGHV3–23	97.22%	Not Classified	del(13q) biallelic
17	62	F	IGHV1–18	97.92%	Not Classified	N
18	71	M	IGHV3–66	97.89%	Not Classified	del(13q) monoallelic
19	34	M	IGHV4–30	97.25%	Not Classified	N
20	47	M	IGHV4–59	97.89%	Not Classified	N
21	77	F	IGHV3–21	97.92%	Not Classified	del(13q) and del(11q)

F, Female; M, Male; del, deletion; N, Normal.

*subset classification according to the ARResT database.

## Results and discussion

**Figure 1:**
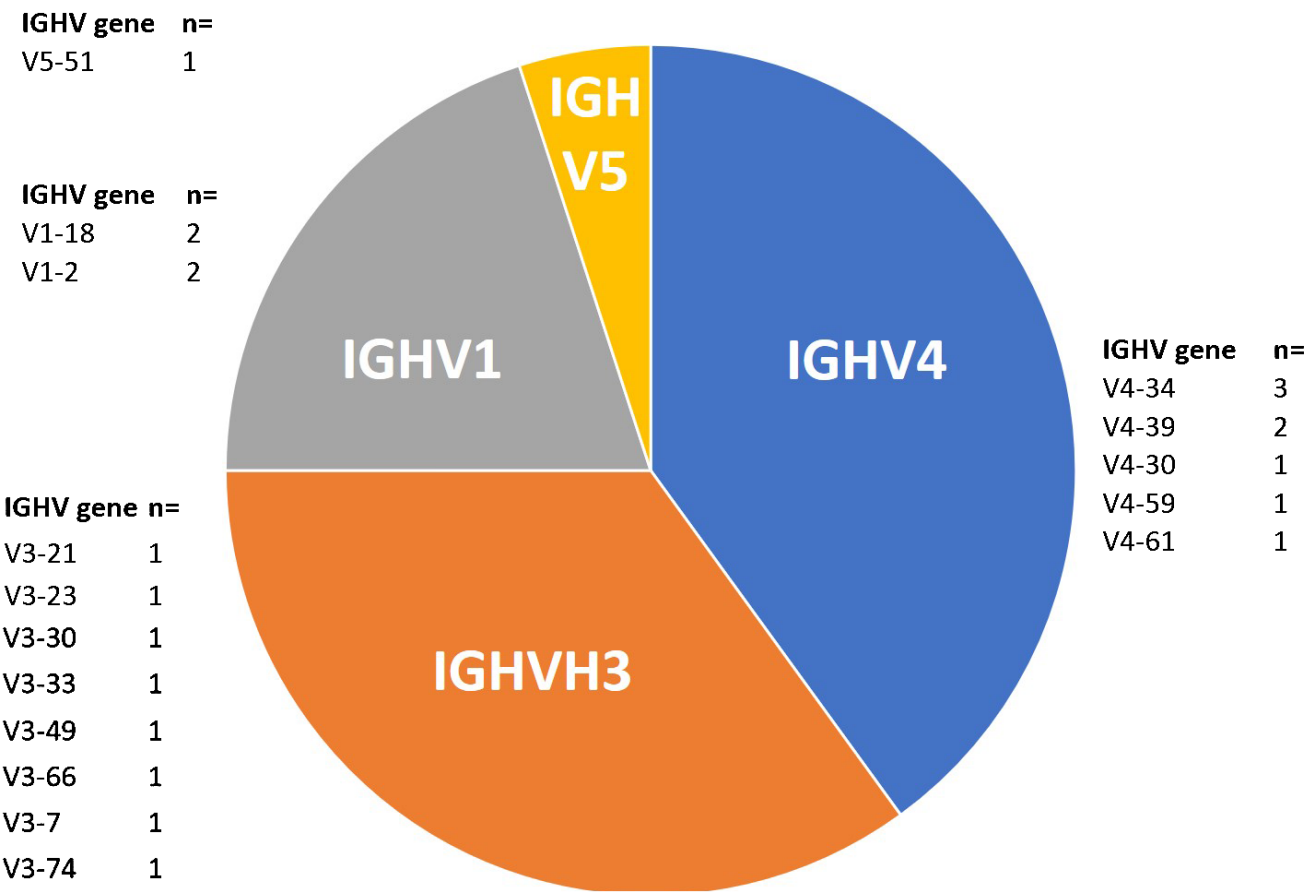
Distribution of IGHV genes among BL-IGHV CLL patients (n=21).

Regarding the distribution of *IGHV* genes, *IGHV4* and *IGHV3* both ranked first with 38% each, followed by *IGHV1* and *IGHV5* presenting 19% and 5%, respectively (**Table 1** and **Figure 1**). Meanwhile, in all the Lebanese CLL population, *IGHV3* ranked first (46.2%) followed by *IGHV4* (22.5%) (9). It should be noted that no *IGHV2* or *IGHV6* cases were detected among the 21 patients with BL-IGHV, this may be due to the small sample size or other specific characteristics related to the BL entity of CLL. Regarding the *IGHV* subgroups in patients with BL-IGHV, IGHV4–34 was the most frequently detected, followed by IGHV4–39, IGHV1–18, and IGHV1–2 (**Figure 1**). Interestingly, none of the BL-IGHV patients in our study was assigned to a BCR-subset, using the ARResT online database (10), which is different from previous studies showing an enrichment in subset #2 (8). In fact, in our previous study only 6% of Lebanese patients with CLL could be assigned to a specific stereotyped subset, which is relatively lower than the data reported from other countries (9), and thus potentially suggesting the presence of population-specific alterations.

Concerning the associated cytogenetic abnormalities evaluated by FISH (fluorescent *in situ* hybridization), 52% of the patients with BL-*IGHV* presented a normal pattern, while 48% presented abnormal results (**Table 1**). Interestingly, all abnormal FISH (n=10) showed a del(13q), either as a single abnormality (n=4) or in association with another anomaly such as del(17p), del(11q) or del(13q) in the second allele; which is different from the data reported by Angotzi et al., where del(13q) was the most frequent anomaly followed by del(11q) and +12, without any single case of del(17p) among their 30 BL-CLL patient. Our data on BL-*IGHV* show that patients are cytogenetically classified into different prognostic group; some patients show normal results and cytogenetic anomalies that are associated with a good prognosis (isolated del(13q)), while other BL-IGHV patients present cytogenetic anomalies associated with a poor prognosis (del(11q) and del(17p)).

It is true that one of the limitations of this study is the small sample size (n = 21), however, as it has already been stated that this subgroup of CLL with a low prevalence, present only around 5% of all CLL cases. Furthermore, we recognize the importance of including data on treatments, responses, and outcomes; however, this information was not available to us initially and some patients are still under the “watch and wait” phase. For these reasons, we did not present data on survival and treatment. However, it would be really interesting to undertake bigger multicentric studies with a large sample size for further data collection and to analyze all genetic and clinical data on BL-*IGHV*-CLL. Indeed, it seems that this minor group of patients with BL-*IGHV* requires a specific approach and personalized treatment options. For this reason, evaluating the response to available and new treatments in BL-IGHV patients is primordial.

In this current work, we focused on the molecular and cytogenetic aspects of patients with BL-*IGHV* CLL. It is true that only 5% of CLL patients belong to this group, but it is important to admit that the status of BL-IGHV (97–97.99%) deserves more attention. We believe that this work is a stepping stone towards a better understanding of the course, prognosis, and therapeutic options of BL-*IGHV* CLL disease.

## Data availability statement

The original contributions presented in the study are included in the article/supplementary material. Further inquiries can be directed to the corresponding author.

## Ethics statement

The studies involving humans were approved by Ethical Committee at Saint Joseph University of Beirut. The studies were conducted in accordance with the local legislation and institutional requirements. The participants provided their written informed consent to participate in this study. Written informed consent was obtained from the individual(s) for the publication of any potentially identifiable images or data included in this article.

## Author contributions

SR: Data curation, Formal analysis, Investigation, Methodology, Writing – original draft. WS: Formal analysis, Investigation, Methodology, Software, Writing – original draft. NA: Formal analysis, Investigation, Methodology, Validation, Writing – original draft. RM: Formal analysis, Investigation, Methodology, Software, Writing – original draft. AC: Conceptualization, Formal analysis, Funding acquisition, Investigation, Methodology, Resources, Supervision, Visualization, Writing – review & editing.
